# *Defb19* regulates the migration of germ cell and is involved in male fertility

**DOI:** 10.1186/s13578-022-00924-1

**Published:** 2022-11-22

**Authors:** Jing Jin, Xiaofeng Li, Mei Ye, Feng Qiao, Hao Chen, Kin Lam Fok

**Affiliations:** 1 School of Biomedical Sciences, Faculty of Medicine, The Chinese University of Hong Kong, Hong Kong, SAR China; 2 Department of Laboratory Medicine, Peking University Shenzhen Hospital, Lianhua Road No. 1120, Futian District, Shenzhen, Guangdong People’s Republic of China; 3 Institute of Reproductive Medicine, Medical School of Nantong University, Nantong, People’s Republic of China; 4 Sichuan University-The Chinese University of Hong Kong Joint Laboratory for Reproductive Medicine, West China Second University Hospital, Chengdu, People’s Republic of China

**Keywords:** β-defensin, Spermatogenesis, Cell migration, Male fertility

## Abstract

β-defensins are small antimicrobial peptides that play essential roles in male fertility. Although several members of the β-defensin family are preferentially expressed in the testis, their involvement in spermatogenesis remains elusive. In this study, we have characterized the expression and function of *Defb19* in mouse testis. Our results showed that *Defb19* is expressed in both Sertoli cells and germ cells. Overexpression of *Defb19* in the 15P-1 Sertoli cell line decreases the expression of cell junction molecules and promotes the matrix adhesion and migration of Sertoli cells. Recombinant DEFB19 and conditioned medium of *Defb19*-overexpressed 15P-1 cells promote the migration of GC2-spd spermatocyte cell line. Knockout of *Defb19* in mouse by CRISPR/Cas9 resulted in male subfertility with testicular and epididymal atrophy. A marked increase in apoptosis and a significant decrease in the sperm count were observed in the KO mice. Together, our study has uncovered an important role of *Defb19* in male fertility by regulating the migration of both the Sertoli cells and the germ cells. Our study has shed new light on the functions of β-defensins in the testis.

**Supplementary Information:**

The online version contains supplementary material available at 10.1186/s13578-022-00924-1.

Spermatogenesis is a complicated process involving mitosis, meiosis and post-meiotic differentiation of germ cells. In rodents, spermatogenesis can be further divided into 12 stages, denoted by I-XII and each stage is defined by a unique composition of differentiating germ cells [1]. During spermatogenesis, the differentiating germ cells migrate from the basal compartment, passing through the blood-testis barrier (BTB) formed by the adjacent Sertoli cells, and reach the adluminal compartment. The differentiating germ cells continue to migrate towards the lumen of the seminiferous tubules while being nursed by the Sertoli cells *via* testis-specific adherens junction known as ectoplasmic specialization (ES) [2].

Germ cell migration also involves cell-matrix interaction [3]. Spermatocyte is known to produce matrix metalloproteinases 2 and 9 that degrade extracellular matrix proteins such as laminin for cell migration [4, 5]. In the basal compartment, the spermatogonia are routinely migrating towards a highly vascularized microenvironment where they reside on the basement membrane enriched in laminin and collagens [6]. Thus, the process of germ cell migration involves both cell-cell adhesion and cell-matrix interaction. Defects in the migration of germ cells perturb spermatogenesis and the arrested germ cells undergo apoptosis.

β-defensin is a family of small and cationic antimicrobial peptides. They are expressed in various tissues and cell types, including immune cells (neutrophils and macrophages), and epithelial cells [7]. A number of β-defensins, both ubiquitously expressed or specifically expressed in the reproductive tract, play essential roles in sperm maturation and sperm functions. Ubiquitous human DEFB1 defenses male fertility by regulating sperm motility and host defense [8]. Mutations in reproductive tract-specific human DEFB126 is associated with impaired sperm-egg interaction and subfertility [9]. Apart from the epididymis, a number of β-defensins are also highly expressed in the testis [10]. However, the physiological function(s) of testicular β-defensins has not been explored. *Defb19* is a member of the β-defensin family specifically expressed in the reproductive tracts. Previous studies showed that, *Defb19* or its human homolog *DEFB119* is robustly expressed by both the Sertoli cells and the germ cells and the expression is the highest among other members of the β-defensin family [7, 11, 12]. Transcriptome analysis revealed the differential expression of DEFB119 in the epididymis of bovine with demonstrated subfertility [13]. However, the physiological function(s) of *Defb19* in the male reproductive tract remains elusive. In this study, we revealed the involvement of *Defb19* in the migration of germ cells. Loss of *Defb19* led to male subfertility.

## Expression profile of *Defb19*

**Fig. 1:**
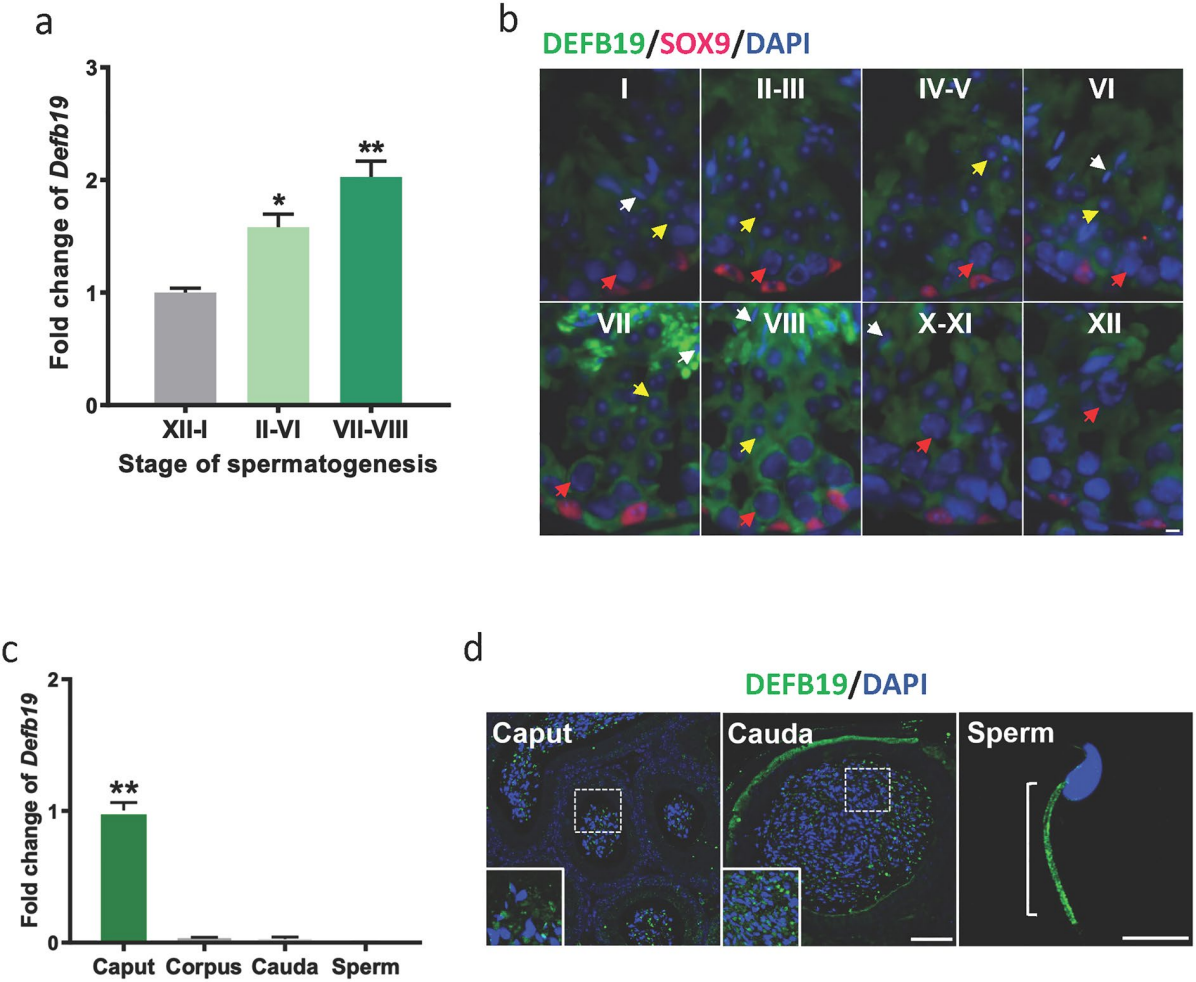
The expression profile of *Defb19* in the male reproductive tract of mice. **a** RT-qPCR analysis comparing the expression of *Defb19* in the indicated stages of spermatogenesis (n = 3 mice each with three technical replicates). GAPDH was used as internal control. **b** Representative images of frozen sections of testis immunofluorescence staining (n = 3 mice) showing the expression of DEFB19 (green) in germ cells and Sertoli cells. SOX9 (red) marks the nuclei of Sertoli cells, DAPI (blue) marks cell nuclei. Red arrowhead indicate spermatocyte, Yellow arrowhead indicate round spermatid, and white arrowhead is spermatid, respectively, Scale bar = 5 μm. **c** RT-qPCR analysis comparing the expression of *Defb19* in three segments, caput, corpus and cauda of epididymis (n = 3 mice each with three technical replicates) and in matured sperm. GAPDH was used as internal control. **d** Representative immunofluorescence staining images of paraffin-embed epididymis section (n = 3 mice) showing the expression of DEFB19 (green), DAPI (blue) marks cell nuclei, Scale bar = 50 μm in the epididymis, scale bar = 10 μm in the sperm. Data are presented as means ± SD, analysed by One-way ANOVA combined Tukey’s multiple comparisons test. * p < 0.05, ** p < 0.01

*Defb19* is known to be expressed in the Sertoli cell in the mouse testis for decades [12]. We first sought to validate the mRNA and protein expression of *Defb19* in multiple spermatogenic stages of the testis, in different segments of the epididymis and on sperm. Our results showed that the mRNA level of *Defb19* was dynamically regulated during the spermatogenic cycle. Specifically, we observed an increase from stage I and the expression peaks at stage VIII (Fig. 1a). We further validated the protein level of DEFB19 by immunostaining and characterized the cell types expressing DEFB19. We co-stained Sertoli cells with SOX9 and differentiated various stages of germ cells by their nuclear morphologies and location within the seminiferous tubules. The signals of DEFB19 were observed in the Sertoli cells, meiotic and post-meiotic germ cells, including round, elongated spermatids and spermatozoa at stage VII to VIII (Fig. 1b). Notably, a more recent study using single-cell RNA sequencing revealed that *Defb19* is expressed in both the Sertoli cells and the germ cells [14] and the types of germ cells with higher expression of *Defb19* are actively undergoing cell movement/migration at stage VIII of the seminiferous tubules, i.e. preleptotene spermatocyte transiting the BTB, and spermatozoa releasing from the lumen. This is in line with our finding that DEFB19 protein is observed in both the germ cells and Sertoli cells in the seminiferous tubules with the observation of the peak expression of *Defb19* at stage VIII.

In the epididymis, the mRNA of *Defb19* was only detected in the caput, but not in the corpus and cauda (Fig. 1c). Of note, similar to other β-defensins that are captured by the sperm during epididymal maturation, we located the DEFB19 signals in matured spermatozoa isolated from the vas deferens and cauda, as well as in the lumen of caput (Fig. 1d). While the matured sperm *per se* did not carry *Defb19* mRNA (Fig. 1c), the signal of DEFB19 was observed at the midpiece of sperm. These results suggest that the signal of DEFB19 in the sperm is generated from the testis and the epididymis and is maintained by the matured sperm.

## Overexpression of *Defb19* alters the cell-adhesion properties of Sertoli cells

**Fig. 2:**
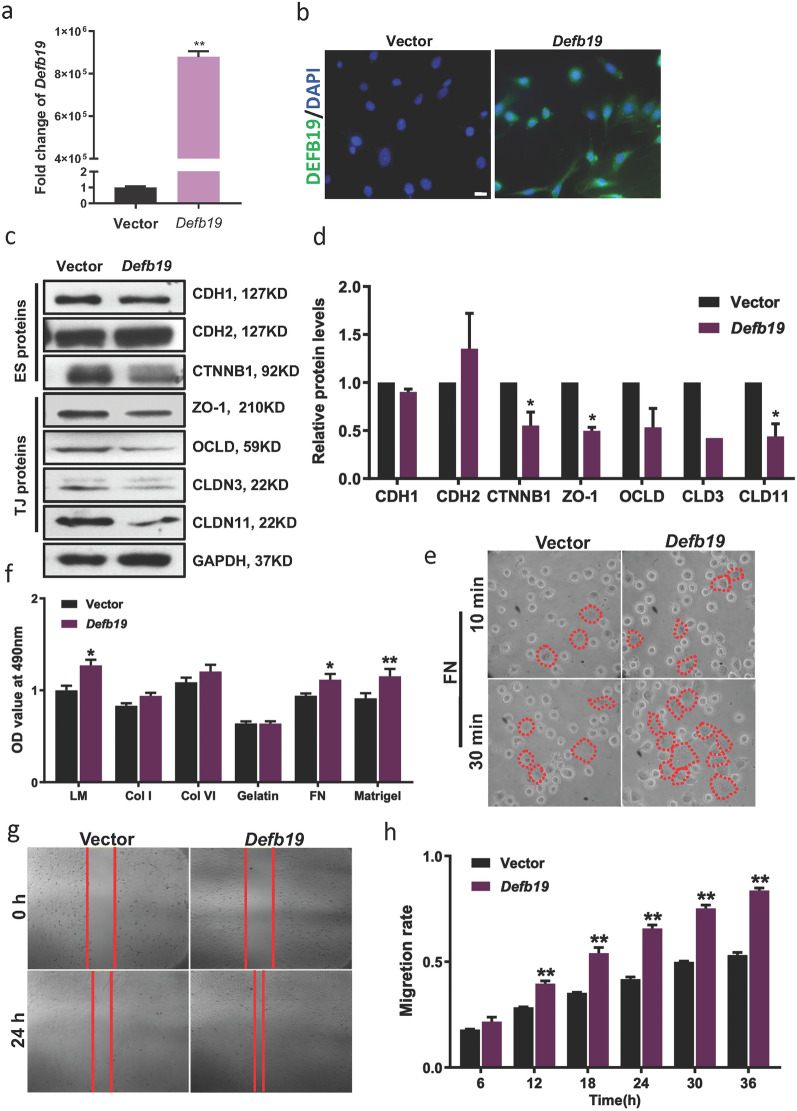
Overexpression of *Defb19* alters cell adhesion and motility of Sertoli cell. **a**, **b** RT-qPCR analysis (**a**) and representative immunofluorescence staining images (**b**) comparing the expression of *Defb19* in *Defb19*- or vector control-transduced 15P-1 Sertoli cell line. DEFB19 was marked by green. DAPI (blue) marks cell nuclei. Scale bar = 20 μm. **c** Representative Western blot result of ES proteins (E-Cadherin - CDH1, N-Cadherin – CDH2 and β-Catenin – CTNNB1) and TJ proteins (ZO-1, Occludin - OCLD, Claudin3 – CLDN3, and Claudin11 – CLDN11) in *Defb19*-overexpressed and vector control transduced 15P-1 lines. GAPDH was used as loading control. **d** Quantification of Western blot results (n = 3 independent experiments). (e) Representative phase contrast images of adhesion assay showing the attachment of indicated 15P-1 lines on fibronectin (FN) at indicated time points. Attached cells were marked by red dashed line. **f** MTS assay quantifying the number of attached cells in adhesion assay (e, n = 3 independent experiments). **g** Representative time-lapse imaging images showing the migration of indicated 15P-1 lines at indicated time points. Red bars indicate the cell boundaries. **h** Quantitation of the migration rate of 15P-1 cells in the time-lapse migration assay at indicated time points (n = 3 independent experiments). Data are presented as means ± SD, analyzed by Student’s t test (**A**–**C**). * p < 0.05, ** p < 0.01

Since *Defb19* was highly expressed at stages VII-VIII during spermatogenesis when the restructure of BTB and break down of apical ES occur, we designed experiments to explore the involvement of *Defb19* in these processes. We have used the mouse Sertoli cell line 15P-1 with no endogenous expression of *Defb19*. *Defb19* was overexpressed in 15P-1 cells (Fig. 2a, b), and tested the expression of the protein components of BTB and ES in *Defb19*-overexpressed 15P-1 by Western blot. We observed a significant decrease in ES proteins (E-Cadherin and β-Catenin) and BTB proteins (ZO-1, Occludin, Claudin3, and Claudin11) (Fig. 2c, d), suggesting that *Defb19* alters the BTB and ES of Sertoli cells. To investigate whether the *Defb19* is associated with the adhesion of Sertoli to the basal membrane, we tested the adhesion ability towards various extracellular matrix proteins by adhesion assay. We observed an increase of adhesion ability on laminin, fibronectin and matrigel in *Defb19*-overexpressed 15P-1 line as compared to vector control transduced line (Fig. 2e, f). Lastly, since remodeling of cell junctional proteins and the alteration of adhesion properties is often associated with cell migration[15], we examined the migration property by wound healing assay. In line with the decreased expression of junctional proteins, the mobility of *Defb19*-overexpressed 15P-1 was significantly increased (Fig. 2g, h). These results suggest that overexpression of *Defb19* in 15P-1 Sertoli cell line alters the BTB and ES and promotes cell adhesion to the basal membrane and cell motility.

## *Defb19* triggers the migration of spermatocyte

**Fig. 3:**
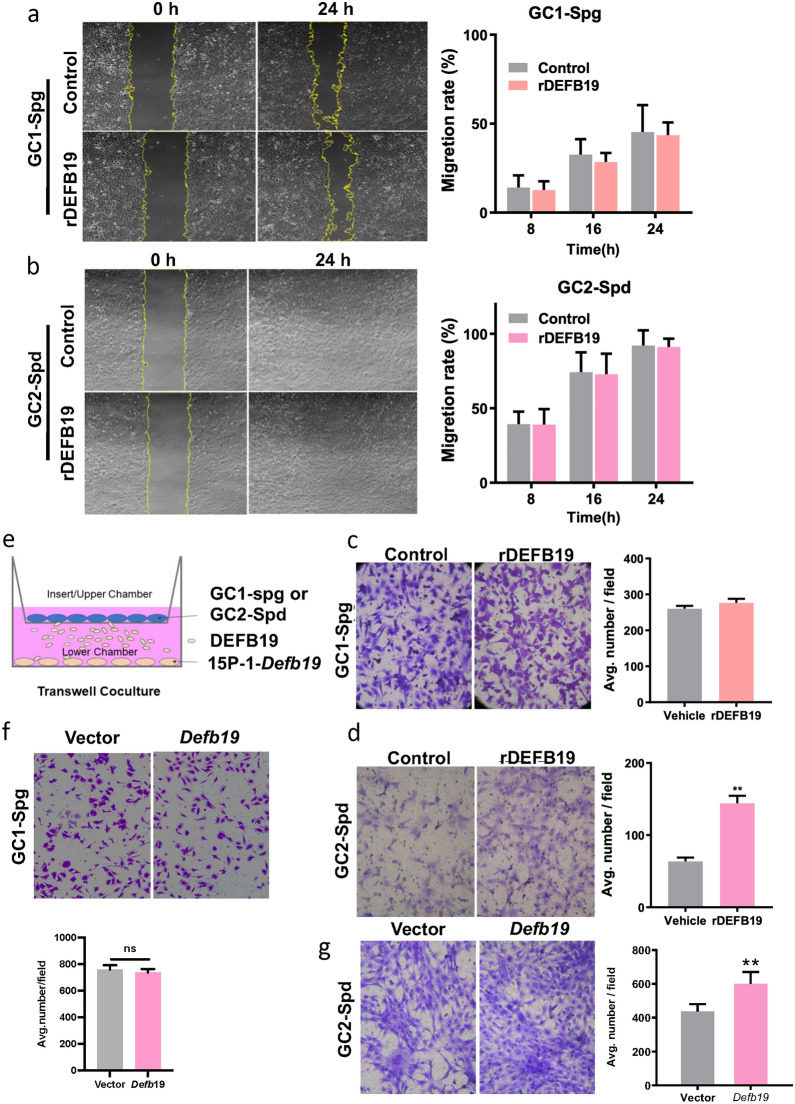
DEFB19 acts as a chemoattractant for spermatocyte migration. **a**, **b** Representative images and corresponding quantification of wound healing assay comparing the closure rate of scratch area both in GC1-Spg (**a**) and GC2-Spd (**b**) treated with 400ng/ml rDEFB19 and vehicle control (n = 3 independent experiments). **c**, **d** Representative images and corresponding quantitative analyses of transwell migration assay comparing the number of GC1-Spg and GC2-Spd (n = 8 independent experiments) migrating through the transwell in response to 400 ng/ml rDEFB19 treatment or vehicle control. **e** Schematic diagram showing the setup of GC1-spg/GC2-Spd and 15P-1 co-cultured transwell migration assay. **f** Representative images showing the migrated GC1-spg co-cultured with *Defb19*-overexpressed or vector control-transduced 15P-1 lines. Corresponding quantitative analyses is shown on the bottom panel (n = 3 independent experiments). **g** Representative images showing the migrated GC2-spd after co-cultured with *Defb19*-overexpressed or vector control-transduced 15P-1 lines. Corresponding quantitative analyses of transwell migration assay is shown on the right (n = 3 independent experiments). Data are presented as means ± SD, analyzed by Student’s t test. ** p < 0.01

The migration of germ cells during spermatogenesis is known to involve Sertoli cell-germ cell interactions and the germ cell-extracellular matrix interaction mediated by matrix metalloproteinases [4, 16]. Hitherto, the widely recognized theory is that the germ cells are tightly attached or in close contact with other germ cells or Sertoli cells [16]. Thus, the movement of developing germ cells is considered passively orchestrated by the direct interactions with these cells. To investigate the effect of *Defb19* on germ cells, we chose GC1-Spg and GC2-Spd, which are cell lines established from spermatogonia and spermatocyte origins respectively [4]. We treated GC1-Spg and GC2-Spd with recombinant DEFB19 (rDEFB19), a matured and active DEFB19 peptide. Interestingly, in contrast to the effect on the motility of the Sertoli cells, we observed that the average closure rate of the scratch area was comparable between groups in both GC1-Spg and GC2-Spd (Fig. 3a, b), suggesting that DEFB19 has a negligible effect on the mobility of germ cells. In the invasion assay that requires the degradation of extracellular matrix, we observed a significant increase in the number of GC2-Spd cells that migrated into the lower chamber side in response to the rDEFB19 treatment but not the vehicle control. Interestingly, no difference was observed in GC1-Spg (Fig. 3c, d), indicating that the spermatocyte but not the spermatogonia respond to the paracrine signal of DEFB19.

To further examine if the effect of DEFB19 was attributed to the paracrine signal of Sertoli cells, we adopted a co-culture system to investigate the chemotactic effect of DEFB19 secreted by the Sertoli cell line. We seeded *Defb19*-overexpressing 15P-1 cells onto the bottom well and cultured the GC1-spg or GC2-Spd lines in the upper chamber (Fig. 3e). Indeed, compared with the vector control transduced 15P-1 line, we observed a significant increase in the migration of GC2-Spd cells but not GC1-spg through the transwell into the lower chamber side containing the *Defb19*-overexpressing 15P-1 line (Fig. 3f, g). These results suggest the paracrine role of DEFB19 secreted by the Sertoli cells in triggering the migration of spermatocytes but not spermatogonia. It should be noted that both migrating germ cells and Sertoli cells express DEFB19. Therefore, although our results showed that the DEFB19 secreted by Sertoli cells triggers the migration of spermatocytes, we could not exclude the possibility that the germ cells-secreted DEFB19 also possesses similar activity. Nonetheless, the induced mobility of spermatocytes could be an active response to initiate the migration towards the adluminal compartment where their further migration is regulated by the cell-cell interaction with Sertoli cells.

## Knockout of *Defb19* leads to male subfertility

**Fig. 4:**
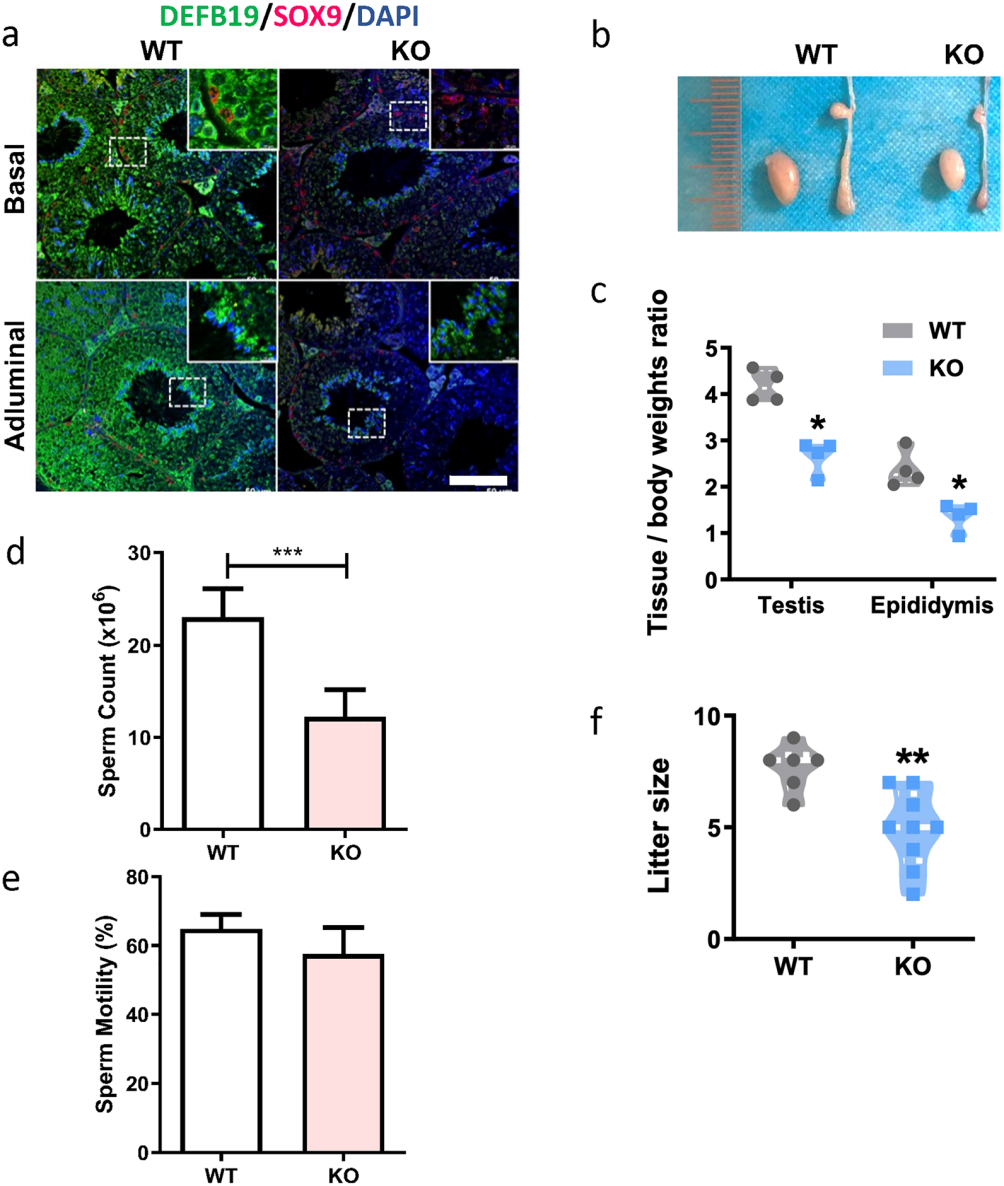
Knockout of *Defb19* leads to male subfertility. **a** Representative immunofluorescence staining images of paraffin-embed testis section showing the protein expression of DEFB19 (green) in WT and KO mice (n = 5 mice). Sertoli cells were marked by SOX9 (red) and nuclei were counterstained with DAPI (blue). Scale bar = 50 μm. b Representative images of the testis and the epididymis of KO and WT mice. **c** Tissue to body weight ratio of the testis and epididymis of KO and WT mice (n = 4 mice). **d**, **e** Number of sperm isolated from the epididymis and vas deferens (**e**, n = 6 mice) and the motility of sperm (**e**, n = 5 mice) in WT and KO mice. **f** Quantitation of the number of litters sired by WT females after cross breeding with KO or WT male. Data are presented as means ± SD, analysed by Student’s t test. * p < 0.05, ** p < 0.01, *** p < 0.001

In the next set of experiments, we sought to examine the effect of DEFB19 on spermatogenesis and male fertility using a loss-of-function model. We knocked out *Defb19* by deleting the exon 1 and 2 that represent the full length of *Defb19* by the CRISPR-Cas9. Wildtype (WT, Defb19^+/+^) and knockout (KO, Defb19^−/−^) mice were obtained by breeding of heterozygous mice (Defb19^+/−^). Immunofluorescence staining confirmed the absence of DEFB19 protein in the KO mice testis compared with their littermate WT control (Fig. 4a). To investigate the effect of the loss of *Defb19* in the male reproductive tract, we examined the testis and epididymis of KO mice and found a significant decrease in both the size and the weight of testis and epididymis of KO (Fig. 4b and c). In line with the decrease in weight, the number of sperm recovered from the cauda epididymis and vas deferens was significantly decreased in KO mice (Fig. 4d). Loss of *Defb19* did not affect the motility of sperm (Fig. 4e). To evaluate the fertility of KO male mice, we bred male KO and its WT littermate to WT females and counted the number of sired pups. The litter size of KO was significantly decreased compared with that in the WT, indicating that knockout of *Defb19* leads to male subfertility (Fig. 4f). These results suggest an important role of *Defb19* in male fertility.

**Fig. 5:**
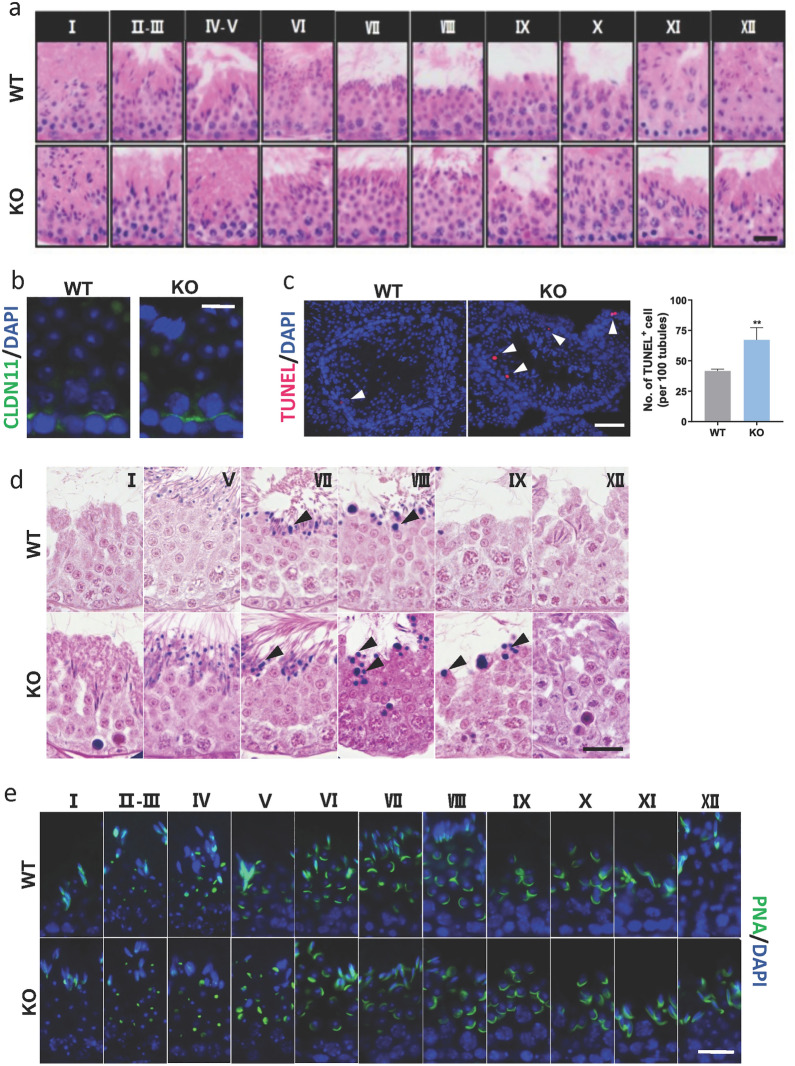
Spermatogenic defects in *Defb19* KO mice. **a** Representative hematoxylin (nuclei, blue) and eosin (cytoplasm, red) staining images of paraffin-embedded testis showing the cell composition and cell morphology at indicated stage of seminiferous tubules of WT and KO mice (n = 6 mice). Scale bar = 20 μm. **b** Representative immunofluorescence staining images showing the expression of claudin 11 (CLDN11, green) in the testis of WT and KO mice. DAPI (blue) marks cell nuclei. Scale bar = 10 μm. **c** Representative images of TUNEL assays showing the apoptotic cells in the WT and KO testes. Corresponding quantitation s shown on the right panel (n = 4 mice). TUNEL (red) marks the apoptotic cell and DAPI (blue) marks cell nuclei. Arrowhead indicates the TUNEL + cells. Scale bar = 50 μm. **d** Representative specific staining images of paraffin-embedded testis showing the RBs at indicated stages of the testis from indicated groups (n = 4 mice). The cytoplasm is pink. The RB is blue (arrowheads). Scale bar = 10 μm. **e** Representative PNA-lectin staining images of paraffin-embedded testis showing the acrosome of developing spermatid from indicated groups (n = 6 mice), PNA (green) marks the acrosome of spermatid and DAPI (blue) marks cell nuclei. Scale bar = 20 μm. Data are presented as means ± SD, analysed by Student’s t test. ** p < 0.01

We further compared the histology of the testis from WT and KO mice since we observed atrophy in the testis of KO mice. To our surprise, no observable difference regarding the morphology and location of different germ cell types were observed across spermatogenesis in the KO mice (Fig. 5a). A comparable expression of claudin 11, a component of the BTB, was also observed in the KO (Fig. 5b), suggesting that the BTB was intact.

**Fig. 6:**
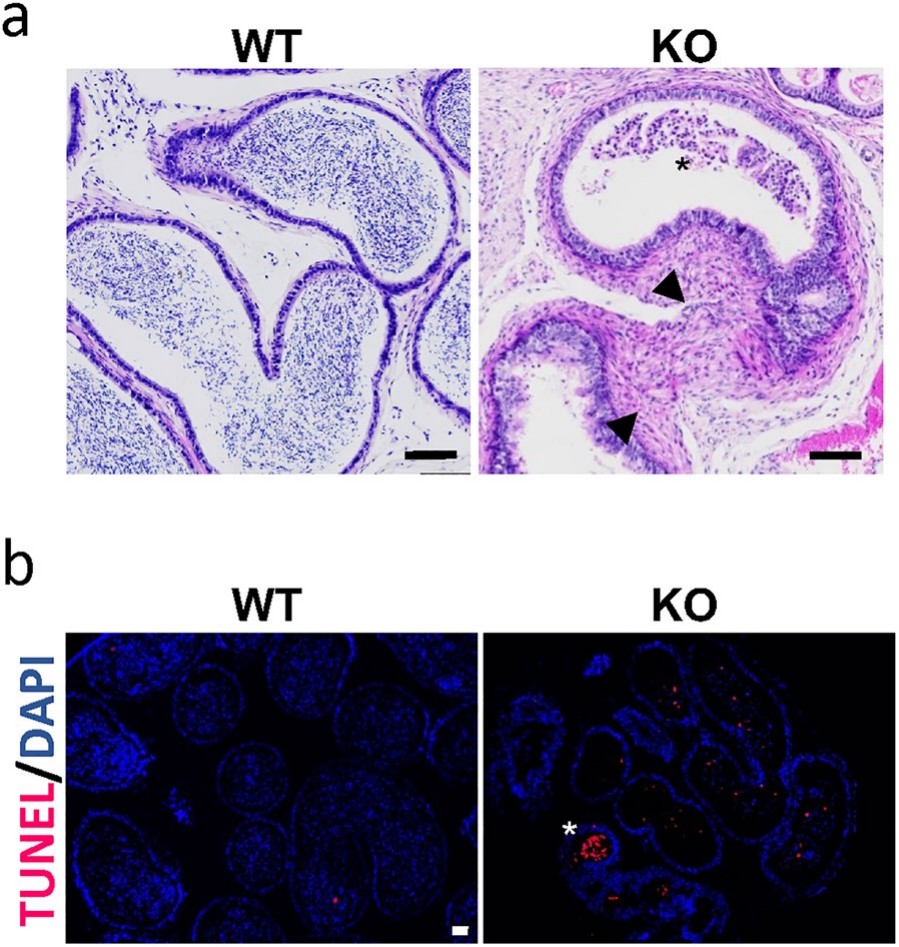
Abnormalities in the epididymis of *Defb19* KO mice. **a** Representative hematoxylin (nuclei, blue) and eosin (cytoplasm, red) staining images of paraffin-embedded epididymis showing the accumulation of non-sperm cells (asterisk) in the lumen of KO mice (n = 3). Fibrosis (arrowhead) associated with tissue atrophy was also noted in the epididymis of KO mice. **b** Representative images of TUNEL assays showing the apoptotic cells in the WT and KO epididymis. TUNEL (red) marks the apoptotic cell and DAPI (blue) marks cell nuclei. Asterisk indicates the TUNEL + cells. **a**, **b** Scale bar = 50 μm

Since we observed the paracrine effect of DEFB19 in germ cell migration in vitro, we sought to examine the migration of germ cells in KO mice. While molecular markers for the migration of germ cells remain to be defined, defects in the germ cell migration are associated with the apoptosis of the germ cell [4, 17]. Thus, we performed the TUNEL assay to compare the apoptosis in WT and KO testis. Interestingly, we found a significant increase in the number of apoptotic germ cells in the seminiferous tubule of KO mice (Fig. 5c). We then investigated the apoptotic body of degenerated germ cells or the residual bodies (RBs) that are formed from the cytoplasm of spermatid during spermiogenesis by methylene blue/azure II stain. Indeed, we observed positive stains near the pachytene spermatocytes at stage VIII. We also observed the positive stains at the lumen of the seminiferous tubules that represent the RBs. Intriguingly, we observed an increase in the number of residual bodies, at stage IX in the KO mice testis compared to that of WT control (Fig. 5d). These results demonstrated that loss of *Defb19* leads to an increase in the apoptosis of meiotic germ cells and dysregulated RBs formation or removal during spermiogenesis, suggesting that the subfertile phenotype may be attributed to dual defects during spermatogenesis. Since the development of acrosomes precedes the formation of RBs, we carried out PNA staining to examine the fine structure of acrosome formation during spermiogenesis in WT and KO mice. We observed comparable staining patterns of the acrosome located at the anterior part of the spermatid nucleus (Fig. 5e), indicating that *Defb19* is not required for acrosome development. In corroboration with the increase in apoptosis in the testis and the decrease in sperm count, we observed the abnormal accumulation of non-sperm cells and a marked increase in apoptosis in the lumen of the epididymis of KO mice (Fig. 6a, b).

The present study is the first to investigate the physiological functions of β-defensins in the testis and in spermatogenesis. While β-defensins are known to play pivotal roles in immune modulation by recruiting immune cells and regulating cytokine secretion [18, 19], our study has revealed the involvement of *Defb19* in regulation germ cell migration and cell junctions independent of the immune modulation of the testis. In the present study, we showed that the spermatocyte but not the spermatogonia respond to the paracrine signal of DEFB19. The induced mobility of spermatocytes could be an active response to initiate the migration towards the adluminal compartment where their further migration is regulated by the cell-cell interaction with Sertoli cells. Defects of which increase the apoptosis in the testis and epididymis associated with male subfertility (Additional file 1).

In summary, we have uncovered the important role of *Defb19* in male fertility by regulating the migration of both the Sertoli cells and the germ cells. The findings of the present study have shed new light on the functional study of β-defensins in the testis that may lead to a new diagnosis and therapy regimen for male infertility.

## Supplementary Information

**Additional file 1.** Materials and methods.

## Data Availability

Not applicable.

## Abbreviations

Defb19: Defensin beta 19
*DEFB119*: Defensin beta 119
SOX9: SRY (sex determining region Y)-box 9
BTB: Blood-testis barrier
ES: Ectoplasmic specialization
Spg: Spermatogonia
Spd: Spermatocyte
WT: Wildtype
KO: Knockout
TUNEL: Terminal deoxynucleotidyl transferase biotin-dUTP nick end labeling
RBs: Residual bodies
