# Supplementary material for: Defb19 regulates the migration of germ cell and is involved in male fertility
Source: Cell Biosci. 2022 Nov 22;12:188. doi: 10.1186/s13578-022-00924-1 (PMC9682749; doi:10.1186/s13578-022-00924-1)
Supplement: Supplementary file 1 — Additional file 1. Materials and methods. [file 13578_2022_924_MOESM1_ESM.docx]

**Materials and Methods**

*Cell culture*

15P-1, GC1-Spg and GC2-Spd cell lines were cultured in DMEM (Thermo 12800017) supplemented with 1:100 diluted Penicillin Streptomycin (Thermo 15140122) and 10% fetal bovine serum (Thermo 10270106) and maintained at 37 °C in the presence of 5% CO_2_ of incubator.

*Lentiviral transduction*

The clone of Defb19 was purchased from a commercially available source and validated by sequencing. The Defb19 was inserted into pLVX-EF1α-IRES-mCherry plasmid using EcoR I and Xba I. Lentiviral particles were prepared by co-transfection of 293FT cells with envelope plasmid (pMD2.G), packaging plasmids (psPAX2) and pLVX plasmid with or without Defb19 insert. Then the target cell lines were transduced with lentivirus and the mCherry+ cells were identified and sorted by the flow cytometry. Stable expression of Defb19 in the transduced lines was confirmed by real time PCR.

*Mice*

*Defb19*-KO mouse model was generated by Cyagen Biosciences Inc. All the mice were maintained in a C57BL/6 background. *Defb19*-KO mice were obtained by crossing heterozygote male mice with heterozygote female mice.

Breeding assay was performed by housing adult KO mice and their WT littermate with WT virgin adult female at ratio of 1 : 2 (M : F). Then the male mice were taken from the breeding cage after breeding a week. When female gave birth to the pups, the pups number was recorded.

*RT-PCR*

The RNA was extracted with RNAiso plus (Takara 9108) according to the manufacturer instructions. Briefly, samples were lysed in RNAiso plus, then mRNA was separated by chloroform (Lab-Scan A3505E) and precipitated by isopropanol (Duksan 859) after centrifugation. Lastly, the pellet was air-dried and dissolved by RT-PCR grade water. The quality and concentration were confirmed by Nanodrop (Spectrophotomter 2000c).

The total amount of 500 ng RNA was used for reverse transcription reaction. Briefly, the reaction mixture was prepared with PrimeScript RT Master Mix (Takara RR360A) and run method was 37℃ 15 min for reverse-transcription, 85℃ 5 sec for inactivating transcriptase, hold at 4℃.

The qPCR mixture was prepared with SYBR Premix (Takara RR420D) and run method was 95°C for 30 sec, 95°C for 5 sec repeating for 45 cycles, 60°C for 5 min. The data was calculated by 2^-△△CT method.

*MTS assay*

The initial seeding density was 1000 cells per well of a 96-well plate. Three hours before each measurement, the medium was discarded and 100 μl fresh culture medium and 10 μl CellTiter Solution were added to the assaying wells, the cells were incubated at 37 °C and 5% CO2 for 3 hr in the dark, the OD value was measured at 490 nm.

*Western blot*

The cells were lysed by RIPA buffer (lab made) and 1:100 diluted protease and phosphatase Inhibitor Cocktail (Hou-Bio 78442) on the ice, then the lysis was centrifuged at 12000 rpm for 30 min. The supernatant was aliquoted and stored at -80°C.

The protein concentration was measured with Protein assay dye reagent concentrate (Bio-Rad 500-0006) according to the manufacture instruction. A total of 40 μg protein was loaded into 10% SDS-PAGE gel for electrophoresis. The run time was adjusted by the molecular weight of target proteins. Then target protein on the gel was transferred to the 0.2 μm pore-size PVDF membrane (Sigma ISEQ00010). The transferring time was according to the molecular weight of target proteins, usually 20~60 min. After transferring, the PVDF membrane was blocked with 5% non-fat milk in TBST (lab made) and immunoblotted with primary antibody which was diluted in 5% milk non-fat TBST at 4°C overnight. Then, the membrane was washed three times with TBST, and then incubated with 1:10000 diluted secondary antibody conjugated with HRP in the 5% milk for 1 hours at room temperature. Lastly, PVDF membrane was washed three times with TBST for 10 min. Signals were detected with Amersham ECL Advance Western Blotting Detection Kit (GE RPN2135) and Super X-film (Fuji Medical) according to the manufacturer instructions. The antibodies and dilution ratio as listed below.

*Adhesion assay*

The 96-well plate was coated with different ECM components and Matrigel and incubated at 4°C overnight. Meanwhile, the cells were starved with 4% FBS for 12 h. The dissociated cells were seeded into a 96-well plate with 20000 cells per well. After incubating 30 min at 37 °C and 5% CO2, the medium of cells was smoothly discarded. The fresh medium containing 10% FBS was added into the cells for recovery for 4 hr. Lastly, the cell medium was discarded and supplemented with 100 μl fresh culture medium and 10 μl CellTiter Solution, the cells were incubated at 37 °C and 5% CO2 for 3 hr in the dark, the OD value was measured at 490 nm.

*Transwell migration assay*

The transwell chambers were placed into a 24-well plate and the chamber was immersed into 2% FBS culture medium. The cells were seeded into the transwell chamber (Corning 3422) at a density of 2x10^4^ cell per chamber and with 2% FBS culture medium. Recombinant DEFB19 (rDEFB19) at various doses, 200 ng/ml, 400 ng/ml, or 600 ng/ml were added into the wells of the 24-well plate. After culturing for 48 hr, the upper chambers were fixed with 1% crystal violet (Sigma C-3886 dissolved in 10% Methanol). After clearing the non-migrated cells with a cotton swab, the chamber membrane was checked and random images of the membrane were taken under microscope. The number of migrated cells per field was calculated.

For co-culture transwell migration assay, the cells were seeded into a 24-well plate at a density of 5x10^4^ cells per well. Then the cells were cultured at 37 °C and 5% CO_2_ for 48 hr and refreshed with 2% FBS medium. The remaining steps were the same as in the Transwell migration assay except the addition of 2% FBS medium containing the 10 μg/ml DEFB19 antibody (Abcam 126867) or Normal goat IgG (Millipore NI02-100UG) as negative control.

*Wound healing assay*

The cells were seeded into a 6-well plate at a density of 106 cells per well. When the cell confluence reached 100%, the cross scratch was made by a 20 μl tip on the bottom of the well with confluent cells. The cells were washed with PBS three times and added 2% FBS medium. Six sites of every cross scratch were tracked and recorded at indicated time points over a period of 24 hr. The migration rate was calculated from that the migrated area was divided by the initial scratch area.

*Immunostaining*

The 5 μm of paraffin-embedded testis sections were made by microtome (Leica RM 2235), after dewaxed, the samples were proceeded to antigen retrieval. Testis were collected and embedded in OCT for cryosectioning. The 5 μm of section were made by Leica cryostat. The samples were permeabilized with 0.25% Triton X-100 (Sigma T9284) and washed with PBS (lab made) three times. Then the non-specific binding sites of samples were blocked with blocking medium (1% BSA (Sigma A3803), 22.52 mg/mL glycine (IBI IB70194) in PBST). The samples were applied to primary antibody which was diluted in 1% BSA in PBST (lab made) in a humidified chamber at 4°C overnight. After washed with PBST three times, the samples were incubated with secondary antibody in 1% BSA in the dark at RT. Then the samples were washed with PBS three times and mounted by the mount medium (Fisher scientific H-1200). The images were taken under the microscopes (Leica TCS SP8 White Light (WLL) Confocal Microscope or Olympus IX83 Inverted Microscope with ZDC). All antibodies are listed as below:

*TUNEL staining*

The 5 μm of paraffin-embedded testis sections were made by microtome (Leica RM 2235), after dewaxed, the samples were incubated with 20 μg/ml Proteinase K solution (Qiagen 19131) at 37°C for 30 min. The samples were washed twice with PBS for 3 min. The mixture of Label Solution and Enzyme Solution was prepared. After samples were applied with TUNEL reaction mixture and incubated in a humidified atmosphere for 60 min at 37°C in the dark, the samples were washed twice with PBS for 3 min and mounted by the mount medium (Fisher scientific H-1200). The images were taken under the microscopes (Leica TCS SP8 White Light (WLL) Confocal Microscope or Olympus IX83 Inverted Microscope with ZDC).
